# Hemodynamics of short-duration light-intensity physical exercise in the prefrontal cortex of children: a functional near-infrared spectroscopy study

**DOI:** 10.1038/s41598-024-66598-6

**Published:** 2024-07-06

**Authors:** Takashi Naito, Koichiro Oka, Kaori Ishii

**Affiliations:** 1 Graduate School of Sport Sciences, Waseda University, 2-579-15 Mikajima, Tokorozawa, Saitama 359-1192 Japan; 2 Organization for the Strategic Coordination of Research and Intellectual Properties, Meiji University, Jinbocho, Chiyoda-Ku, Tokyo 101-8301 Japan; 3 Faculty of Sport Sciences, Waseda University, 2-579-15 Mikajima, Tokorozawa, Saitama 359-1192 Japan

**Keywords:** Light-intensity physical activity, Children, Adolescents, Prefrontal cortex, Functional near-infrared spectroscopy, Oxygenated hemoglobin, Health care, Risk factors

## Abstract

Identifying the types of exercise that enhance cerebral blood flow is crucial for developing exercise programs that enhance cognitive function. Nevertheless, few studies have explored the amount of light-intensity, short-duration exercises that individuals can easily perform on cerebral blood flow, particularly in children. We examined the effects of these exercises on the hemodynamics of the prefrontal cortex (PFC) using functional near-infrared spectroscopy. Participants comprised 41 children (aged 12.1 ± 1.5 years, 37% female) who engaged in seven light-intensity exercises, with each movement performed in two patterns lasting 10 or 20 s. Changes in oxygenated hemoglobin (oxy-Hb) levels at rest and during exercise were compared using analysis of covariance, with sex and age as covariates. Significant increases in oxy-Hb were observed in multiple regions of the PFC during all forms of exercise (including dynamic and twist stretching [66.6%, 8/12 regions, η^2^ = 0.07–0.27], hand and finger movements [75.0%, 9/12 regions, η^2^ = 0.07–0.16], and balance exercises (100.0%, 6/6 regions, η^2^ = 0.13–0.25]), except for static stretching with monotonic movements. This study implies that short-duration, light-intensity exercises, provided that they entail a certain degree of cognitive and/or physical demands, can activate the PFC and increase blood flow.

## Introduction

Cognitive functions refer to all the “intellectual functions” performed in the cerebrum, including memory, thinking, understanding, judgment, language, and calculation. Physical activity is defined as any bodily movement produced by skeletal muscles that requires energy expenditure^1^, and exercise, a subset of physical activity, is a planned, structured, and repetitive bodily movement aimed at improving or maintaining physical fitness^2,3^. Several studies have demonstrated that exercise improves cognitive function^4–10^. In addition to exercise, preventing excessive sitting is essential, as sedentary behavior has been shown to reduce cognitive function^11–13^. However, 81% of children worldwide are not sufficiently physically active, which includes inadequate participation in exercise^14^. Moreover, longitudinal studies have revealed that decreased physical activity and increased sedentary behavior during childhood and adolescence^15–17^. Therefore, concerns remain regarding adverse effects on healthy brain development and cognitive function in children.

Numerous studies have demonstrated the positive effects of exercise on executive function, which represents a higher cognitive function. Executive function is the ability to select appropriate actions to achieve a certain target^18^. The prefrontal cortex (PFC), located in front of the neocortex, is involved in this particular function^19^. Executive functions included the following: (1) inhibition (reducing behavioral impulsivity and maintaining attention), (2) working memory (temporarily retaining and processing information necessary for a task), and (3) cognitive flexibility (responding to changes in a situation)^20^. The development of executive functions during childhood is essential because these functions evolve from infancy to early adulthood and serve as the foundation for building self-control and smooth interpersonal relationships with others. Second, in addition to influencing academic performance, interpersonal relationships, obesity, depression, and problematic behaviors during childhood and adolescence, executive function is linked to health status in adulthood^21^.

In a study on children, Hillman et al. found that a single 20-min session of moderate-intensity aerobic exercise increased inhibitory function^22^, and a nine-month after-school exercise intervention program increased executive function^23^. A meta-analysis of 36 randomized controlled trials (14 acute and 22 habitual exercises) investigating the association between exercise and executive function revealed that both acute and habitual exercise improved executive function in children. The findings indicated small effect sizes for inhibition and cognitive flexibility, and moderate effect sizes for working memory^24^. To date, most studies examining the effects of exercise interventions on cognitive function have focused on moderate-to-vigorous-intensity exercise. Several recent studies on adults and older participants have reported that light-intensity exercise, such as slow walking and cycling, improve executive function^25–28^.

Based on human and animal experiments, the mechanisms by which exercise improves cognitive and executive functions are believed to involve exercise-related improvements in cerebral blood flow, structural changes in the brain, suppression of neuroinflammation, and promotion of neurogenesis^29,30^. Regarding cerebral blood flow, studies demonstrated that cerebral blood flow improves during and immediately after moderate-to-high-intensity acute^31,32^ and habitual^33–35^ aerobic exercises. However, studies have yet to investigate changes in cerebral blood flow during light-intensity exercise. To the best of our knowledge, no previous studies have focused specifically on children. To utilize the knowledge that exercises improve brain function in real-world settings, such as schools (e.g., between classes, during class, and in homerooms), considering the acceptability of the content^36,37^ and the utilization of short duration is crucial. Light-intensity exercise is considered an effective way to achieve this goal. Light-intensity exercise may help improve executive function, whereas increasing light-intensity physical activity may improve obesity markers and cardiovascular health in children^38–41^.

Thus, the present study investigated the effects of short-term and light-intensity exercises, such as stretching, which can be easily performed on the spot without the need for special equipment, on PFC hemodynamics by measuring changes in cerebral blood flow using functional near-infrared spectroscopy (fNIRS). Furthermore, because the effects on cerebral blood flow differ, depending on the type of exercise and duration of movement^39,42^, in this study, seven types of exercise were performed with two patterns of 10 and 20 s per movement. The present study also examined differences in the effects of the type of exercise and duration of each movement on the hemodynamics of the PFC. In such cases, developing exercise programs that are effective in improving executive function that children can easily engage in at school, home, and elsewhere may be beneficial.

## Results

### Number of participants and analyzed fNIRS data

A total of 41 participants were included in the analysis. After excluding data that could not be analyzed owing to body motion artifacts based on the assessment criteria, 34 (82.9% male, 23 participants) and 37 (90.2% male, 23 participants) participants were eligible for analysis in patterns 1 (10 s) and 2 (20 s), respectively. Table 1 summarizes the characteristics of the participants.

**Table 1:** Characteristics of all participants and those analyzed in Patterns 1 and 2.

	All		Pattern 1		Pattern 2	
	N	%	N	%	N	%
Age, mean ± SD, years	12.1 ± 1.5		12.1 ± 1.4		12.1 ± 1.4	
Sex						
Male	26	63	23	68	25	68
Female	15	37	11	32	12	32
Grade						
5th Grade	10	24	8	24	8	22
6th Grade	2	5	1	3	2	5
7th Grade	16	39	14	41	16	39
8th Grade	7	17	6	18	6	17
9th Grade	6	15	5	15	5	14
Dominant hand						
Right	38	93	31	91	34	92
Left	3	7	3	9	3	8

*SD* standard deviation.

### Ratings of perceived exertion (RPE)

The RPE of each exercise category, which was assessed in the participants using the Borg scale (minimum 6 to maximum 20), was less than “11 (light)” for all responses. The mean RPE for the whole group was 7.3 ± 1.8, with a score of 7 corresponding to “very very light,” and a score of 9 corresponding to “very light” on the Borg scale. All exercise events used in this study were light-intensity.

### Hemodynamic changes in the PFC

#### Comparison between resting and light-intensity exercise (Pattern 1)

In the 10-s pattern of one movement, only middle PFC (M-PFC) (F [1, 64] = 4.34; *P* < 0.05) in event A exhibited a significant increase in the oxygenated hemoglobin (oxy-Hb) Z-score during exercise compared to that at rest. In Event B, no significant differences were observed between the resting and exercise conditions in any region. For event C, the oxy-Hb Z-score significantly increased during exercise in the M-PFC (F [1, 64] = 21.71; *P* < 0.001) and left PFC (L-PFC) (F [1, 64] = 14.45; *P* < 0.001). Additionally, in event D, the oxy-Hb Z-score significantly increased during exercise in the M-PFC (F [1, 64] = 4.86; *P* < 0.05) and L-PFC (F [1, 64] = 5.74; *P* < 0.05). In event E, the oxy-Hb Z-score significantly increased during exercise in the L-PFC (F [1, 64] = 8.39; *P* < 0.01). During events F and G, the oxy-Hb Z-scores increased significantly in all the PFC regions. Specifically, each value was in the right PFC (R-PFC) (F [1, 64] = 5.40; *P* < 0.05), M-PFC (F [1, 64] = 6.11; *P* < 0.05), and L-PFC (F [1, 64] = 8.53; *P* < 0.01) for event F, while in event G, significant increases were observed in the R-PFC (F [1, 64] = 10.80; *P* < 0.01), M-PFC (F [1, 64] = 13.45; *P* < 0.001), and L-PFC (F [1, 64] = 14.55; *P* < 0.001) (Table 2).

**Table 2:** Comparison of changes in oxy-Hb Z-scores at rest and during light-intensity exercise (Pattern 1).

Event (ID-Seconds, Name)	Region	Oxy-Hb (mM・mm)				Oxy-Hb (Z-score)				F	*P*	η2
		Rest		Exercise		Rest		Exercise				
		Mean	SD	Mean	SD	Mean	SD	Mean	SD			
A-10 Upward Stretch	R-PFC	0.03	0.10	0.06	0.19	− 0.09	0.68	0.09	1.26	0.56	0.455	0.01
	M-PFC	0.01	0.13	0.10	0.23	− 0.25	0.68	0.25	1.21	4.34	0.041*	0.06
	L-PFC	0.02	0.11	0.06	0.15	− 0.15	0.84	0.15	1.14	1.49	0.227	0.02
B-10 Shoulder Stretch	R-PFC	0.00	0.09	0.01	0.21	− 0.04	0.54	0.04	1.33	0.12	0.734	0.00
	M-PFC	0.03	0.19	0.07	0.25	− 0.07	0.88	0.07	1.13	0.31	0.579	0.00
	L-PFC	0.01	0.11	0.03	0.11	− 0.12	1.02	0.12	1.00	1.00	0.321	0.01
C-10 Elbow Circles	R-PFC	− 0.01	0.18	0.06	0.24	− 0.16	0.85	0.16	1.14	1.71	0.195	0.03
	M-PFC	− 0.06	0.14	0.14	0.21	− 0.50	0.69	0.50	1.04	21.71	< .001*	0.25
	L-PFC	− 0.03	0.12	0.09	0.13	− 0.42	0.87	0.42	0.97	14.45	< .001*	0.18
D-10 Trunk Twist	R-PFC	− 0.01	0.14	0.04	0.30	− 0.10	0.62	0.10	1.29	0.60	0.441	0.01
	M-PFC	− 0.04	0.15	0.06	0.21	− 0.27	0.80	0.27	1.13	4.86	0.031*	0.07
	L-PFC	− 0.02	0.12	0.06	0.15	− 0.29	0.84	0.29	1.09	5.74	0.020*	0.08
E-10 Washing Hands	R-PFC	0.01	0.13	0.05	0.25	− 0.10	0.63	0.10	1.28	0.70	0.407	0.01
	M-PFC	0.00	0.09	0.09	0.25	− 0.22	0.48	0.22	1.32	3.26	0.076	0.05
	L-PFC	− 0.02	0.10	0.06	0.12	− 0.34	0.89	0.34	1.01	8.39	0.005*	0.12
F-10 Thumb & Pinky	R-PFC	− 0.02	0.16	0.06	0.12	− 0.28	1.10	0.28	0.84	5.40	0.023*	0.08
	M-PFC	− 0.02	0.13	0.09	0.20	− 0.29	0.72	0.29	1.17	6.11	0.016*	0.09
	L-PFC	− 0.02	0.12	0.06	0.11	− 0.34	0.98	0.34	0.93	8.53	0.005*	0.12
G-10 Single Leg Balance	R-PFC	− 0.06	0.16	0.08	0.17	− 0.38	0.90	0.38	0.98	10.80	0.002*	0.14
	M-PFC	− 0.10	0.19	0.07	0.18	− 0.42	0.96	0.42	0.89	13.45	< .001*	0.17
	L-PFC	− 0.08	0.13	0.07	0.17	− 0.42	0.78	0.42	1.04	14.55	< .001*	0.18

Covariates included sex and age.

*R-PFC* right prefrontal cortex, *M-PFC* central prefrontal cortex, *L-PFC* left prefrontal cortex, *SD* standard deviation, oxy-Hb, oxygenated hemoglobin.

*Significant difference between the mean Z-scores at rest and during exercise (*P* < .05).

#### Comparison of between rest and light-intensity exercise (Pattern 2)

For the pattern of 20 s per movement in event A, the oxy-Hb Z-score significantly increased only in the R-PFC (F [1, 70] = 6.98; *P* < 0.05) during the exercise. In event B, no significant differences were observed in any region during the exercise. In event C, the oxy-Hb Z-score significantly increased during the exercise in the M-PFC (F [1, 70] = 5.15; *P* < 0.05). In events D and E, the oxy-Hb Z-scores significantly increased during the exercise in all the PFC regions. Specifically, for event D, each value in the R-PFC (F [1, 70] = 15.71; *P* < 0.001), M-PFC (F [1, 70] = 19.59; *P* < 0.001), and L-PFC (F [1, 70] = 25.88; *P* < 0.001) demonstrated a significant increase. Similarly, for event E, a significant increase was observed in the R-PFC (F [1, 70] = 7.15; *P* < 0.001), M-PFC (F [1,70] = 14.16; *P* < 0.001), and L-PFC (F [1, 70] = 5.04; *P* < 0.05). In event F, the oxy-Hb Z-score significantly increased during the exercise in the R-PFC (F [1, 70] = 12.78; *P* < 0.001) and L-PFC (F [1, 70] = 9.64; p < 0.01) regions. In event G, the oxy-Hb Z-score significantly increased during the exercise in all the PFC regions, including the R-PFC (F [1, 70] = 17.55; *P* < 0.001), M-PFC (F [1, 70] = 10.96; *P* < 0.01), and L-PFC (F [1, 70] = 22.93; *P* < 0.001) regions (Table 3).

**Table 3:** Comparison of changes in oxy-Hb Z-scores at rest and during light-intensity exercise (Pattern 2).

Event (ID-Seconds, Name)	Region	Oxy-Hb (mM mm)				Oxy-Hb (Z-score)				F	*P*	η2
		Rest		Exercise		Rest		Exercise				
		Mean	SD	Mean	SD	Mean	SD	Mean	SD			
A-20 Upward Stretch	R-PFC	− 0.03	0.20	0.09	0.20	− 0.30	0.95	0.30	0.98	6.98	0.010*	0.09
	M-PFC	0.01	0.23	0.13	0.34	− 0.20	0.79	0.20	1.16	2.86	0.095	0.04
	L-PFC	− 0.04	0.25	0.04	0.30	− 0.13	0.90	0.13	1.10	1.26	0.265	0.02
B-20 Shoulder Stretch	R-PFC	0.01	0.13	0.04	0.16	− 0.09	0.90	0.09	1.11	0.59	0.446	0.01
	M-PFC	0.01	0.27	0.11	0.23	− 0.19	1.06	0.19	0.93	2.61	0.111	0.04
	L-PFC	− 0.01	0.13	0.05	0.18	− 0.17	0.83	0.17	1.14	2.07	0.154	0.03
C-20 Elbow Circles	R-PFC	0.03	0.14	0.06	0.25	− 0.08	0.72	0.08	1.23	0.48	0.490	0.01
	M-PFC	− 0.02	0.15	0.13	0.36	− 0.26	0.53	0.26	1.28	5.15	0.026*	0.07
	L-PFC	0.00	0.12	0.06	0.22	− 0.16	0.70	0.16	1.23	1.75	0.190	0.02
D-20 Trunk Twist	R-PFC	− 0.03	0.13	0.14	0.21	− 0.42	0.69	0.42	1.10	15.71	< .001*	0.18
	M-PFC	− 0.06	0.21	0.18	0.27	− 0.44	0.80	0.44	1.01	19.59	< .001*	0.20
	L-PFC	− 0.04	0.12	0.14	0.17	− 0.52	0.71	0.52	1.00	25.88	< .001*	0.27
E-20 Washing Hands	R-PFC	− 0.02	0.12	0.07	0.18	− 0.30	0.74	0.30	1.15	7.15	0.009*	0.09
	M-PFC	− 0.04	0.13	0.13	0.25	− 0.40	0.63	0.40	1.15	14.16	< .001*	0.16
	L-PFC	− 0.01	0.14	0.07	0.16	− 0.26	0.93	0.26	1.03	5.04	0.028*	0.07
F-20 Thumb & Pinky	R-PFC	− 0.03	0.13	0.10	0.17	− 0.39	0.84	0.38	1.02	12.78	< .001*	0.15
	M-PFC	0.01	0.23	0.10	0.20	− 0.21	1.08	0.21	0.90	3.23	0.077	0.04
	L-PFC	− 0.04	0.12	0.07	0.16	− 0.35	0.81	0.35	1.08	9.64	0.003*	0.12
G-20 Single Leg Balance	R-PFC	− 0.06	0.14	0.09	0.15	− 0.45	0.87	0.45	0.94	17.55	< .001*	0.20
	M-PFC	−0.08	0.15	0.06	0.20	− 0.36	0.82	0.36	1.05	10.96	0.001*	0.13
	L-PFC	− 0.09	0.16	0.08	0.16	− 0.50	0.88	0.50	0.88	22.93	< .001*	0.25

Covariates included sex and age.

*Significant difference between the mean Z-scores at rest and during exercise (*P* < .05).

*R-PFC* right prefrontal cortex, *M-PFC* middle prefrontal cortex, *L-PFC* left prefrontal cortex, *SD* standard deviation, *oxy-Hb* oxygenated hemoglobin.

#### Comparison of change rate of oxy-Hb z-score between Patterns 1 and 2

No significant difference was observed in the rate of change of the oxy-Hb Z-score between 10 and 20 s per movement pattern in any of the PFC regions (Table 4).

**Table 4:** Comparison of oxy-Hb Z-score changes between Pattern 1 and 2.

Event (ID, Name)	Region	Pattern 1 ΔZ-score		Pattern 2 ΔZ-score		F	*P*
		Mean	SD	Mean	SD		
A Upward Stretch	R-PFC	0.18	1.36	0.60	1.34	0.80	0.376
	M-PFC	0.50	1.31	0.39	1.49	0.03	0.867
	L-PFC	0.30	1.68	0.26	0.82	0.01	0.922
B Shoulder Stretch	R-PFC	0.08	1.47	0.18	1.16	0.04	0.836
	M-PFC	0.14	1.25	0.38	1.55	0.26	0.612
	L-PFC	0.24	1.49	0.34	1.46	0.03	0.860
C Elbow Circles	R-PFC	0.32	0.99	0.16	1.47	0.14	0.708
	M-PFC	1.00	1.10	0.51	1.46	0.84	0.362
	L-PFC	0.85	1.22	0.31	1.52	0.88	0.352
D Trunk Twist	R-PFC	0.19	1.53	0.84	1.51	0.36	0.550
	M-PFC	0.53	1.74	0.89	1.39	0.11	0.745
	L-PFC	0.57	1.32	1.04	1.40	0.64	0.426
E Washing Hands	R-PFC	0.21	1.50	0.60	1.40	0.31	0.582
	M-PFC	0.44	1.51	0.81	1.29	0.25	0.616
	L-PFC	0.68	1.40	0.52	1.51	0.07	0.786
F Thumb & Pinky	R-PFC	0.55	1.68	0.77	1.41	0.14	0.711
	M-PFC	0.59	1.40	0.41	1.26	0.14	0.714
	L-PFC	0.68	1.25	0.69	1.28	0.00	0.977
G Single Leg Balance	R-PFC	0.76	1.30	0.90	1.30	0.09	0.762
	M-PFC	0.83	1.53	0.72	1.13	0.05	0.818
	L-PFC	0.85	1.35	0.99	1.27	0.08	0.778

The covariates were sex, age, and oxy-Hb Z-scores at rest.

*R-PFC* right prefrontal cortex, *M-PFC* middle prefrontal cortex, *L-PFC* left prefrontal cortex, *SD* standard deviation, *oxy-Hb* oxygenated hemoglobin.

## Discussion

The present study measured cerebral blood flow dynamics in the PFC during light-intensity exercise and investigated the differences in their effects depending on the exercise type and duration of movement. To the best of our knowledge, this is the first study to use fNIRS to examine changes in cerebral blood flow in the PFC of children during various light-intensity exercises. The main finding of the present study was that the oxy-Hb level significantly increased during exercise compared to that at rest. Based on the type of exercise, an increase in blood flow to the PFC was observed even during light-intensity exercise of short duration. Specifically, a less significant increase in oxy-Hb was observed during static stretching with monotonous movements, whereas more significant increases were observed during static stretching with twisting movements, dynamic stretching, hand exercises, and balance exercises, which require greater physical and/or cognitive loads. A comparison between 10 and 20 s per movement revealed no significant difference in the rate of change in oxy-Hb from rest to exercise in any of the events used in this study.

The mean increase in oxy-Hb level during the exercise among the light-intensity exercise events used in this experiment amounted to 0.12 mM-mm for Pattern 1 and 0.15 mM-mm for Pattern 2. In a previous study examining changes in the contralateral prefrontal oxy-Hb in patients with stroke using fNIRS, no significant increase was observed when they repeatedly held a handgrip with their healthy hand for 10 s at 20% maximum voluntary contraction (MVC) compared with that at rest. However, significant increase of 0.02 mM-mm was observed when performed at 50% and 80% MVC. Moreover, a 0.02 mM-mm increase was observed when performed at 50% MVC^42^. In a previous study using a bicycle ergometer to measure changes in oxy-Hb during cycle exercise, oxy-Hb increased significantly by approximately 0.1 mM-mm during normal cycling at moderate intensity and during a dual-task in which computational problems were performed during cycle exercise compared with that at rest^43^. However, direct comparisons between the findings of this study and those of prior research are not feasible due to variations in the processing of fNIRS data across studies, as well as differences in the calculation of changes in oxy-Hb, which were presented as relative values.

The short-duration, light-intensity exercise used in this study increased cerebral blood flow, which is an indicator of PFC activation. This suggests that short-duration, light-intensity exercises can be included in exercise programs to improve children's executive function. However, it is unclear whether the increase in oxy-Hb observed in this study effectively improved executive function. Nevertheless, an interesting finding of this study was that the hemodynamics of the PFC varied across exercise types among light-intensity exercises. In the future, based on the findings of this study, it will be necessary to examine whether these exercises have a beneficial effect on executive function using light-intensity exercise programs that combine movements found to be effective in increasing PFC hemodynamics.

Event A consisted of a simple static stretching movement in which the participants stretched upward with their folded hands, and Event B consisted of a simple static stretching movement in which the participants stretched their shoulder muscles by pulling one arm toward their chest. In event A, both patterns displayed a significant increase in oxy-Hb in only one region of the PFC. In event B, no significant increase was observed in any region, suggesting that these events were generally less effective in increasing blood flow in the PFC. Previous studies using yoga have demonstrated that complex poses with higher cognitive loads increase PFC activity compared to simple poses^44^. In the present study, simple postural stretching also imposed the minimal cognitive load necessary for motor coordination and attentional control in the PFC. Therefore, the light-intensity exercise used in this experiment may have limited PFC activation.

Event C involved dynamic stretching in which the participants placed their fingers on their shoulders and rotated their elbows widely. Event D involved stretching with a horizontal plane movement in which the participants stretched their hands forward and twisted their upper bodies to hold them in place. Compared with monotonous static stretches A and B, the aforementioned stretches require more physical effort and cognitive load for motor control. These factors may have contributed to the significant increase in oxy-Hb levels during exercise in multiple regions in both events. Reduced movement times were significantly effective for event C, whereas prolonged movement times were effective for event D. Although different from the index used in the present study and from the perspective of improving exercise performance, such as muscle power and speed, it is recommended that dynamic stretching be performed as quickly as possible. If the speed is not specified, it is optimal to perform it 10–15 times per event because an increase in the number of times leads to a decline in exercise performance^45^. In this experiment, dynamic stretching in Event C was performed relatively slowly to minimize the generation of body movement noise caused by head movements. Although the speed varied somewhat among the participants, the number of movements was approximately 8–9 movements in Pattern 1 and 16–18 in Pattern 2, which exceeded the optimal number of movements described earlier for Pattern 2. Further verification is needed to clarify the optimal number and speed of dynamic stretches required to increase the PFC activity.

Event E was an exercise in which participants rubbed their hands together, whereas Event F involved a complex finger movement exercise. In event E, Pattern 1 was less effective in activating the PFC, whereas Pattern 2 was effective in all regions. For Event F, both patterns were effective. Although the intensity of the exercise was extremely low in both events, the effects of exercise on the PFC were still significant. One factor that may have contributed to the increase in PFC activity is the strong association between hand and finger movements and the PFC. Previous studies in older adults demonstrated that hand and finger manipulation exercises increase blood flow to the PFC and improve executive function^46^. Regarding the improvement in cerebral blood flow to the PFC, the results of Event F in the present study are consistent with previous findings. Although event E was a relatively monotonous movement that did not require as much hand dexterity as event F and was considered to have a reduced cognitive load, blood flow significantly increased in all PFC regions in Pattern 2.

Event G involved a one-legged balance exercise performed with both eyes open. Both patterns exhibited moderate to high effect sizes in all PFC regions. The cerebellum plays a vital role in maintaining balance, whereas the PFC plays a role in coordinating movements and focusing attention on maintaining balance^47^. A previous study focusing on young adult males demonstrated that standing on one leg increased PFC activity and cognitive performance^45,48^. In the present study, controlling postural sway in a one-legged stance increased PFC activity in both patterns, suggesting that this resulted in a significant increase in blood flow.

A comparison of the rate of change in oxy-Hb between Patterns 1 (10 s) and 2 (20 s) in terms of the time per movement demonstrated no significant difference. This result refutes the idea that a prolonged duration is effective in increasing PFC activity. The optimal exercise duration may vary, depending on the type of exercise, necessitating further studies to investigate this possibility. The activation of the R-PFC, M-PFC, and L-PFC differed, depending on the type of exercise and duration of movement. Many brain regions, including the motor cortex, cerebellum, and parietal lobe, are involved in exercise planning and execution. Additionally, the PFC is involved in exercise planning, decision making, and attention control. Generally known functions of the brain include emotional processing and spatial recognition in the R-PFC, decision-making, attentional control, and motivation in the M-PFC, and language processing and logical thinking in the L-PFC. However, in the present study, we did not identify a specific pattern of activation in each PFC region. Because previous studies on PFC activation by region during exercise are limited, elucidating the factors and mechanisms underlying the differences in PFC activation by region between the different events and patterns observed in this study will be an issue for future studies.

The present study has several limitations. Firstly, the age range of participants was limited to 10–15 years old. Expanding the age range and increasing the number of participants are needed to examine whether similar results can be obtained in children and adolescents of all ages, considering that the PFC continues to grow until early adulthood. Additionally, it is desirable to obtain maturity data, such as peak height velocity, and use it as an adjustment variable for statistical analysis because of the influence of sex and differences in the degree of growth and development of children of the same sex. Secondly, the number of exercise events and movements used in the experiments was limited because of the constraints of the fNIRS measurements and experimental time. Although limited by not maximizing head movements during fNIRS usage, further validation is necessary to generalize the findings of this study, which indicate low PFC activation during simple static stretching and increased activation with higher physical and cognitive loads, especially for exercise events that were not included in this study. Thirdly, this study did not consider individual physical fitness levels, daily physical activity levels, exercise habits, body mass index, or cognitive abilities. However, cerebral hemodynamics may differ depending on these factors. Finally, this study observed an increase in oxy-Hb levels in the PFC even after brief, light-intensity exercise, suggesting increased blood flow and possibly neural activity in the PFC. However, the impact of these results on executive function has not been verified and requires further investigation in future studies.

## Methods

### Participants

The participants in this study included 41 healthy children from the fifth grade of elementary school to the third year of junior high school. The participants were students from a public junior high school in Tokyo who provided consent for study cooperation from the school principal (14 students), and were recruited using snowball sampling (27 students). Written informed consent to participate in the study was obtained from both the parents and children. Written informed consent was obtained from the person for using images in an online open-access publication. The present study was conducted with approval of the Waseda University Ethics Review Procedures Concerning Research with Human Subjects (approval number: 2021–344). All experiments were performed in accordance with relevant guidelines and regulations.

### Procedure

The experiment was conducted in a conference room at a public facility and in a classroom at the study partner school. Before starting the experiment, the researchers confirmed the participants’ names, ages, gender, grades, and handedness. We then explained the light-intensity exercise to the participants and the precautions to be taken during the measurement (avoiding moving the head as much as possible, not making quick movements, and not holding one’s breath or straining). At that time, we physically performed all exercises as in the actual event and asked the participants for their RPE for each exercise using the Borg scale^49^. Subsequently, an fNIRS device (OEG-16H; Spectratech Inc., Yokohama, Japan) was attached to the head of the individual in a sitting position, and sensor calibration was conducted. After confirming that the equipment was accurately calibrated, the participants began the light-intensity exercise.

### Light-intensity exercise

**Figure 1:**
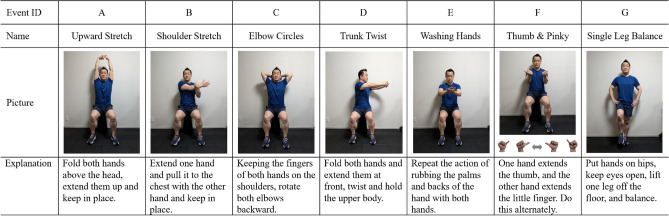
List of light-intensity exercise events and methods used in the experiment.

Seven types of light-intensity exercise (A–G) were performed (Fig. 1). When selecting the exercise types, we focused on two primary considerations: (1) The exercises should be of light intensity and simple enough for children with low motor skills to perform easily in real-world settings (e.g., during a short break in the classroom or performed during class time); and (2) the exercise should have as little head movement as possible to minimize body movement noise during fNIRS measurements to eliminate the effect of increased blood flow due to head tilt. Thus, the exercises used in the present study did not include running or jumping movements; sagittal plane movements, such as flexion of the trunk and head forward or backward; or frontal plane movements, such as tilting the trunk and head laterally. Light-intensity exercises were selected from a list of commonly practiced static and dynamic stretching exercises^50,51^. In addition, as previous studies have shown that hand and balance exercises activate the prefrontal cortex^46,48,52^, these exercises were also included. All events were performed while seated, except for the standing balance exercise, to minimize body movement noise.

**Figure 2:**
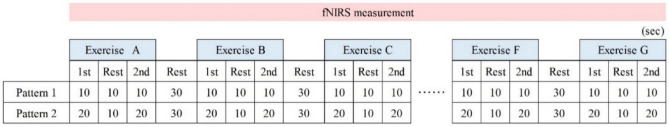
Experimental protocol. The order of patterns (1 and 2) and events (A–G) was randomized.

To investigate the effects of different exercise durations on cerebral blood flow, we created two patterns: one with a 10-s movement pattern (Pattern 1) and the other with a 20-s movement pattern (Pattern 2). Static stretching is recommended for 15–60 s per movement^53^. However, continuous static stretching for ≥ 30 s may temporarily reduce muscle strength^54^ and as the purpose of the present study was not to improve flexibility, but to improve practicality in educational settings, we adopted this particular duration. To eliminate the order effect of events (A–G) and patterns (1 and 2), the order of execution was randomized for all participants. Videos were created in advance for each participant, depicting the exercises in the order in which they were to be performed. The participants performed light-intensity exercises while watching a monitor placed at their eye level with a 10-s rest between the first and second movements. Before moving on to the next event, the participants took a 30-s break, gazing at the naturalistic image on the monitor. After completing the first exercise pattern, the participants rested for 5 min in a seated position. The fNIRS device was then recalibrated and the second pattern was performed (Fig. 2).

### fNIRS

Functional near-infrared spectroscopy (fNIRS) is an imaging technique used to measure changes in cerebral blood flow by irradiating the cranium with near-infrared light, which is highly permeable to tissues in living organisms, such as the skin, bone, and muscle. This imaging modality measures the changes in the concentrations of oxy-Hb and deoxygenated hemoglobin (deoxy-Hb) in the blood. As near-infrared light can only penetrate to a depth of approximately 2 cm from the surface of the head, fNIRS cannot measure changes in blood flow in the deep cerebral cortex. Although its spatial resolution is inferior to that of functional magnetic resonance imaging (fMRI), fNIRS is less sensitive to body motion noise and can measure brain activity in a natural state with fewer restrictions on body position.

**Figure 3:**
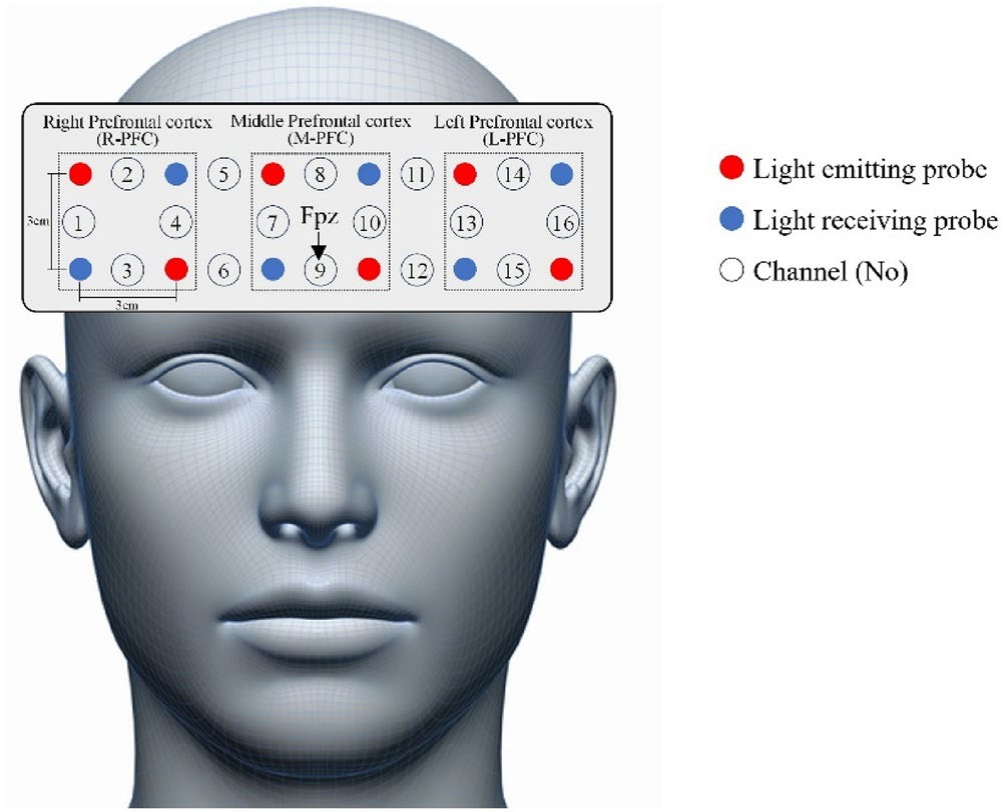
Attachment points and channel locations of the functional near-infrared spectroscopy probes.

In this study, we measured the PFC hemodynamics during light-intensity exercise and at rest using an fNIRS system. The device has six light emitters and six light receivers, with 16 channels. The near-infrared wavelengths were 770 and 840 nm and the distance between the light-emitting and light-receiving sections was 3 cm. The electrodes were placed such that the center of the lower sensor row overlapped with the Fpz, referring to the electrode arrangement of the international 10–20 method for electroencephalogram measurements (Fig. 3). The sampling rate was set to 6.10 Hz (0.08102 s/1 sample), and changes in the concentrations of oxy-Hb, deoxy-Hb, and total hemoglobin in the PFC were measured. The change in oxy-Hb is the most sensitive indicator of regional cerebral blood flow^55^, hence the present study employed the oxy-Hb parameter to indicate cerebral blood flow in the PFC.

### fNIRS data processing

The fNIRS data were preprocessed using software installed on the measurement device. First, all measured data were processed using the hemodynamic separation method^56^ to eliminate the increase in skin blood flow resulting from exercise^57^, which is not a brain-derived signal. To eliminate artifacts caused by breathing and heartbeats, this study applied a high-pass filter at 0.01 Hz and low-pass filter (0.3 Hz) along with bandpass processing to eliminate artifacts caused by breathing and heartbeats. Owing to the potential interference of body movements, the present study examined the data of each participant who received pretreatment and differentiated them based on the waveforms of oxy-Hb and deoxy-Hb The data criteria were as follows: (1) data used when oxy-Hb and deoxy-Hb were in opposite phases; (2) data used when deoxy-Hb was stable near zero; (3) data used when oxy-Hb and deoxy-Hb were in the same phase; and (4) if the proportion of deoxy-Hb was lower than that of oxy-Hb, it was adopted; if the waveform of deoxy-Hb swung significantly compared with that of oxy-HB, the data were rejected. Next, the data to be analyzed were downloaded in CSV format and converted to Excel files using Microsoft Excel 2019 (Microsoft Corp., Redmond, WA, USA). The mean oxy-Hb values for each channel were calculated between 0 and 5 s before the start of the exercise task (at rest) and during the exercise task (10 or 20 s after the start of the exercise task) for each event (A–G) and pattern (1 and 2). Moreover, we averaged the first and second values because each pattern was performed twice for each event. With reference to a previous study^58^, we then divided channels 1, 2, 3, and 4 into three regions: the right PFC (R-PFC); channels 7, 8, 9, and 10 into the M-PFC; and channels 13, 14, 15, and 16 into the left PFC (L-PFC). We calculated the mean oxy-Hb levels at rest and during the exercise for each region. Finally, the mean oxy-Hb values calculated for each pattern were converted to Z-scores. Next, the average oxy-Hb values for each channel were calculated for each exercise event during the 5 s before the start of the exercise task (at rest) and during the exercise task (10 or 20 s after the start of the exercise task).

### Statistical processing

Analysis of covariance (ANCOVA) was used to compare the oxy-Hb Z-scores at rest and during exercise for each domain (R-PFC, M-PFC, and L-PFC) of Patterns 1 and 2. Covariates included sex and age. To examine whether a difference existed in the rate of change in oxy-Hb between Patterns 1 and 2, we also included sex, age, and oxy-Hb Z-score at rest as covariates and compared them using ANCOVA. The significance level was set at *P* < 0.05. All statistical analyses were conducted using IBM SPSS (version 29.0; (SPSS Inc., Armonk, NY, USA).

## Conclusion

In the present study, we investigated the impact of short and light-intensity exercises on the hemodynamics of the PFC. The findings revealed a significant increase in oxy-Hb levels in multiple regions of the PFC during exercise, compared to the resting state. However, no significant change in oxy-Hb levels was observed during static stretching, which is a monotonous movement. These results imply that even brief periods of light-intensity exercises, such as dynamic stretching, balance exercises, and hand exercises, which require a certain amount of physical effort and/or cognitive demands, can enhance PFC activity and blood flow. Further research is needed to explore whether short-duration, light-intensity exercises can enhance cognitive function in children.

## Data Availability

The datasets generated and/or analyzed in the current study are not publicly available due to ethical considerations; however, they are available from the corresponding author upon reasonable request.
